# Geographical variations of the associations between health interventions and all-cause under-five mortality in Uganda

**DOI:** 10.1186/s12889-019-7636-x

**Published:** 2019-10-22

**Authors:** Betty B. Nambuusi, Julius Ssempiira, Fredrick E. Makumbi, Jürg Utzinger, Simon Kasasa, Penelope Vounatsou

**Affiliations:** 1 Swiss Tropical and Public Health Institute, P.O. Box, CH-4002 Basel, Switzerland; 2 University of Basel, P.O. Box, CH-4003 Basel, Switzerland; 3 Makerere University School of Public Health, New Mulago Hospital Complex, P.O. Box 7072, Kampala, Uganda

**Keywords:** Bayesian proportional hazards geostatistical models, Demographic and health survey, Geographical variations, Interventions, Sub-national scale, Uganda, Under-five mortality

## Abstract

**Background:**

To reduce the under-five mortality (U5M), fine-gained spatial assessment of the effects of health interventions is critical because national averages can obscure important sub-national disparities. In turn, sub-national estimates can guide control programmes for spatial targeting. The purpose of our study is to quantify associations of interventions with U5M rate at national and sub-national scales in Uganda and to identify interventions associated with the largest reductions in U5M rate at the sub-national scale.

**Methods:**

Spatially explicit data on U5M, interventions and sociodemographic indicators were obtained from the 2011 Uganda Demographic and Health Survey (DHS). Climatic data were extracted from remote sensing sources. Bayesian geostatistical Weibull proportional hazards models with spatially varying effects at sub-national scales were utilized to quantify associations between all-cause U5M and interventions at national and regional levels. Bayesian variable selection was employed to select the most important determinants of U5M.

**Results:**

At the national level, interventions associated with the highest reduction in U5M were artemisinin-based combination therapy (hazard rate ratio (HRR) = 0.60; 95% Bayesian credible interval (BCI): 0.11, 0.79), initiation of breastfeeding within 1 h of birth (HR = 0.70; 95% BCI: 0.51, 0.86), intermittent preventive treatment (IPTp) (HRR = 0.74; 95% BCI: 0.67, 0.97) and access to insecticide-treated nets (ITN) (HRR = 0.75; 95% BCI: 0.63, 0.84). In Central 2, Mid-Western and South-West, largest reduction in U5M was associated with access to ITNs. In Mid-North and West-Nile, improved source of drinking water explained most of the U5M reduction. In North-East, improved sanitation facilities were associated with the highest decline in U5M. In Kampala and Mid-Eastern, IPTp had the largest associated with U5M. In Central1 and East-Central, oral rehydration solution and postnatal care were associated with highest decreases in U5M respectively.

**Conclusion:**

Sub-national estimates of the associations between U5M and interventions can guide control programmes for spatial targeting and accelerate progress towards mortality-related Sustainable Development Goals.

## Background

Under-five mortality (U5M) is an important indicator of the social and economic development of a specific country or a smaller administrative unit [1]. The government of Uganda has made progress in reducing the U5M; yet, it is still unacceptably high. According to the data obtained from the Demographic and Health Surveys (DHS), Uganda’s U5M declined from 137 deaths per 1000 live births in 2006 to 90 in 2011 [2, 3]. Over the same 5-year period, the coverage of health interventions improved country-wide. For example, the percentage of children receiving vitamin A supplements in the past 6 months increased from 36 to 57%. The percentage of children with fever 2 weeks prior to a survey who were given artemisinin-based combination therapy (ACT) increased from a mere 3% in 2006 to 69% in 2011. Coverage of other health interventions improved as well [2, 3].

Despite this progress at the national level, there are remarkable sub-national variations in U5M and health intervention coverage. The lowest U5M rate (56 deaths per 1000 live births) and the highest (152 deaths per 1000 live births) were observed in Kampala and the North-East regions, respectively. The coverage of vitamin A supplements was 30% in Central 1, compared to 74% in the North-East. Skilled delivery varied from 31% in the North-East to 93% in Kampala. Such disparities may be associated with the observed discrepancies in regional mortality rates and thus need to be investigated at a regional scale.

In Uganda, there is a paucity of studies estimating the relation between U5M and health interventions at a local scale. In addition, most studies that assessed the relationship between U5M and health interventions were not carried out at the national scale. For example, Bacillus Calmette Guerin (BCG) vaccination was associated with a lower rate of death among children aged between one and 5 years in a community-based prospective cohort study in the eastern part of Uganda [4]. A volunteer community health worker child promotion model was related to child mortality declines in rural South-West Uganda [5].

Household surveys provide a suitable source of national data to monitor progress of U5M and implemented interventions. A national DHS was conducted in Uganda in 2011, which provided the most reliable mortality and interventions data with national coverage. To date, a few national studies in Uganda used DHS data to quantify the relationship between health interventions and child mortality. However, these studies assessed only one category of child health interventions; namely, vaccinations [6]. Furthermore, previous research in other settings has used DHS data to evaluate effects of health interventions on U5M at the global [7] and sub-continental [8] scales. However, former studies did not take into account geographical variations in the coverage of health interventions that may influence U5M patterns [9]. To our knowledge, analyses quantifying associations between health interventions and U5M at a sub-national scale have not been carried out. Sub-national estimates of the geographical distribution of the effects of health interventions can guide control programmes to choose and implement most important interventions at a local scale.

The aim of the present study was to estimate geographically varying associations of health interventions (i.e., control interventions against malaria, water, sanitation and hygiene (WASH), reproductive health, breastfeeding, vaccinations, micronutrient supplementation and treatments) with U5M at the national and sub-national scale to identify interventions associated with the largest reduction in mortality at a sub-national scale, and to estimate hotspots of the U5M in Uganda. Bayesian geostatistical Weibull proportional hazards models were applied to DHS data and spatially varying covariates were introduced to assess the effects of interventions at local scale. The models were adjusted for socio-demographic, environmental and climatic factors. Bayesian kriging was used to estimate hotspots of U5M. Results assist control programmes to implement locally adapted interventions, and therefore reduce regional variation in all-cause U5M.

## Methods

### Country profile

Uganda is situated across the equator in East Africa. The country is bordered by the Democratic Republic of the Congo in the West, Kenya in the East, Rwanda in the South-West, Tanzania in the South and Sudan in the North. Uganda is a land-locked country with a surface area of 241,000 Km^2^. The country is divided into 15 regions, which are further partitioned into 116 districts. The population is approximately 44 million people; about half of the population are younger than 15 years, while children below the age of 5 account for approximately 20% [10].

### Design and study setting

All-cause child mortality data were obtained from women’s birth histories, available in the 2011 DHS, which was carried out between May and December, 2011. A representative sample of 10,086 households was selected for the 2011 DHS, using a stratified two-stage cluster design. In the first stage, 404 clusters were selected from a list of clusters for the 2009/2010 Uganda National Household Survey. The second stage involved selecting households from a complete listing of households in each cluster. Overall, 8674 women aged 15–49 years who were either permanent residents of the households or visitors who slept in the households the night before the survey were eligible to be interviewed on characteristics regarding their children. Mortality data were collected on 7878 children representing the number of children born in the period of 5 years preceding the date of the survey.

### Data and sources

#### Health interventions

The The DHS captures data relating to a number of health interventions, including malaria, micronutrients intake and treatments, the latter depending on whether drugs were taken in the previous night of the survey, 7 days, 2 weeks or 6 months prior to the survey. Such coverages may not reflect the extent of intervention utilization in the 5 years preceding the survey. Thus, to obtain representative estimates of intervention coverages for the period of 5 years preceding the 2011 DHS, we averaged health intervention coverages of the 2006 and 2011 DHS. The 2006 DHS collected data on malaria control interventions different from those in the 2011 DHS, that is, households with at least one insecticide-treated net (ITN), U5 sleeping under an ITN and indoor residual spraying (IRS). For consistency, interventions of the 2009 Uganda Malaria Indicator Survey (MIS) were utilized since they matched with those in the 2011 DHS.

Health interventions considered in this paper comprise of malaria, WASH practices, reproductive health, breastfeeding, vaccinations, micronutrients supplementation and treatments of diseases. Coverage of health interventions was generated at the cluster level [7] because data on various interventions such as the vaccination status of dead children are not reported at an individual level in the DHS. Data at clusters were used to obtain intervention coverages at regions.

Data on malaria interventions were collected by means of household questionnaires and included use and ownership of ITN and IRS. Standard guidelines of the Roll Back Malaria (RBM) were followed in the generation of malaria intervention coverage indicators [11]. The ITN use indicators derived in this analysis comprised the percentage of children U5 and the percentage of the population who slept under an ITN the night preceding the survey and the percentage of ITN used by the population in a household the previous night. The indicator on IRS coverage was generated as the percentage of households sprayed in the past 12 months. ITN ownership indicators included the percentage of households with at least one ITN, the percentage of households with one ITN for every two people and the percentage of the population with access to an ITN within their household. WASH interventions included the percentage of households with an improved source of drinking water, the percentage of households with improved sanitation facilities and the percentage of households with both water and soap/detergent at hand washing places.

Data on the coverage of reproductive health, breastfeeding, vaccinations, micronutrients supplementation and treatment interventions were collected from all eligible women using a pretested questionnaire. The questionnaire comprised reproductive health interventions (the percentage of married women using any family planning method, percentage of pregnant mothers receiving antenatal care (ANC) from a skilled provider, the percentage of pregnant women making four or more ANC visits during their entire pregnancy, the percentage of women who received intermittent preventive treatment for malaria during pregnancy (IPTp), the percentage of births that took place with the assistance of a skilled provider and the percentage of newborns receiving first postnatal checkup from a skilled provider within 2 days after delivery, breastfeeding (the percentage of infants who started breastfeeding within 1 h of birth and the percentage of infants exclusively breastfed during the first 6 months after birth), vaccinations (the percentage of the last-born child fully protected against neonatal tetanus, the percentage of children vaccinated with BCG and measles, the percentage of children with complete vaccination of DPT and polio), micronutrients supplementation (the percentage of children receiving vitamin A supplements, the percentage of children receiving iron supplements in the past 7 days and the percentage of children living in households with iodized of salt) and treatments of diseases (the percentage of children with symptoms of acute respiratory infections (ARIs) who took antibiotics, the percentage of children with diarrhoea given fluid from oral rehydration solution (ORS) sachets or recommended home fluids (RHF), the percentage of children with diarrhoea given zinc sulphates, the percentage of children with fever during the 2 weeks prior to the survey and took ACT and those dewormed in the past 6 months).

Interventions with coverage ≥95% and those lacking sufficient coverage (< 5%) within the regions were excluded from the analysis due to lack of variation in estimating their relation with mortality. These were the percentage of households sprayed with IRS in the past 12 months (%H_IRS, 7%), the percentage of pregnant mothers receiving ANC from a skilled provider (ANC provider, 95%), the percentage of children living in households with iodized salt (iodized salt; 99%) and the percentage of children with diarrhoea given zinc sulphates (zinc; 2%). Table 1 provides a list of health interventions assessed in the study.

**Table 1:** Health interventions, Uganda DHS 2006, 2009 and 2011

Intervention	Description of the intervention
Malaria	
%H_IRS	Percentage of households sprayed with Indoor Residual Spraying (IRS) in the past 12 months
%H_1ITN	Percentage of households with atleast one ITN
%H_1ITN2	Percentage of households with atleast one ITN for every two people
%P_ITNA	Percentage of population with access to an ITN within their household (Percentage of the population that could sleep under an ITN, if each ITN in the household were used by up to two people)
%P_ITNS	Percentage of the population in a household that slept under an ITN the previous night of the survey
%P_ITN5	Percentage of children under 5 years in a household who slept under an ITN the previous night of the survey
%P_ITNU	Percentage of existing ITNs used by the population in a household the previous night of the survey
WASH	
Improved water	Percentage of households with improved source of drinking water
Improved sanitation	Percentage of households using improved sanitation facilities
P_wsoap	Percentage of households with soap or detergent and water at hand washing place
Reproductive health	
Family planning	Percentage of married women using any family planning method
ANC provider	Percentage of pregnant mothers receiving ANC from a skilled provider
4+ ANC visits	Percentage of pregnant women making four or more ANC visits during their entire pregnancy
IPTp	Percentage of women who received intermittent preventive treatment for malaria during pregnancy
Skilled delivery	Percentage of births that took place with the assistance of a skilled provider
Postnatal care	Percentage of newborns receiving first postnatal checkup from a skilled provider within 2 days after delivery
Breastfeeding	
Within 1 h	Percentage of infants who started breastfeeding within 1 h of birth
Exclusive	Percentage of infants exclusively breastfed during the first 6 months after birth
Vaccinations	
Tetanus toxoid	Percentage of last-born child fully protected against neonatal tetanus
BCG	Percentage of children vaccinated against BCG
DPT	Percentage of children with complete vaccination of DPT
Polio	Percentage of children with complete vaccination of polio
Measles	Percentage of children vaccinated against measles
Micronutrients	
VitaminA_sup	Percentage of children receiving vitamin A supplements in the past 6 months
Iron_sup	Percentage of children receiving iron supplements in the past 7 days
Iodized salt	Percentage of children living in households with iodized of salt
Treatments	
Antibiotics	Percentage of children with ARIs symptoms who took antibiotics
ORS or RHF	Percentage of children with diarrhoea given fluid from oral rehydration solution (ORS) sachets or recommended home fluids (RHF)
Zinc	Percentage of children with diarrhoea given zinc sulphates
ACT	Percentage of children with fever during the 2 weeks prior to the survey and took artemisinin-combination therapy (ACT)
Deworming	Percentage of children given deworming medication in the past 6 months

#### Environmental and climatic factors

Environmental and climatic factors were obtained from remote sensing sources and aggregated at the cluster level. Temporal predictors such as land surface temperature (LST), rainfall and normalized difference vegetation index (NDVI) were averaged for the entire year of 2011. Land cover types were provided in 17 categories according to the International Global Biosphere Programme (IGBP) classification scheme and re-grouped into three categories, that is, urban, forest and crops. Distance to permanent water bodies was calculated based on the water category of the land cover data. Table 2 contains a list of environmental and climatic factors together with their spatio-temporal resolutions and data sources.

**Table 2:** Remote sensing data sources^a^

Source	Data type	Temporal resolution	Spatial resolution
MODIS/Terra^b^	LST^l^	8 days	1 km
MODIS/Terra^b^	NDVI^m^	16 days	1 km
U.S. Geological Survey-Earth Resources Observation Systems (USGSS)	Rainfall	10 days	8x8km^2^
Shuttle Radar Topographic Mission (SRTM)	Altitude	na	1 × 1 km^2^
MODIS,IBGD type	Land cover Water bodies	na	0.5 × 0.5km^2^
Global Rural and Urban Mapping project	Urban Rural extent	na	1 × 1 km^2^

*na* Not applicable; Land cover groups (forest, crops, urban); ^a^Land cover data accessed in June 2011 and other data accessed in November 2013; ^b^Moderate Resolution Imaging Spectroradiometer (MODIS)/Terra, available at: http://modis.gsfc.nasa.gov/; ^l^Land surface temperature (LST) day and night; ^m^Normalized difference vegetation index

#### Demographic and socioeconomic factors

Demographic and socioeconomic proxies, including maternal (education, literacy, residence, age at birth, early pregnancy termination, number of children born and working status) and child (sex, birth order, birth interval and mode of delivery) characteristics were incorporated in the analysis at an individual level and were captured using a household questionnaire. The household asset score was aggregated at the cluster level and considered in the analysis as a socioeconomic proxy for households’ socioeconomic status.

### Statistical analysis

A Bayesian geostatistical proportional hazards model assuming a baseline Weibull hazard function was fitted to quantify the associations between health interventions’ coverage and U5M, and to identify the most important interventions. The models were fitted to child-specific deaths and censoring times. Environmental, climatic, demographic and socioeconomic factors were included in the model as potential confounders. Spatial correlation between clusters was modelled by a Gaussian process with a covariance matrix measuring correlation between any pair of clusters by an exponential function of the distance between them. Our model assumed that the relation between health interventions and mortality varied across regions by including spatially varying coefficients to capture the interventions effect. Spatial dependence in the interventions’ effects was modelled by region-specific random effects assuming conditional autoregressive prior distributions.

To identify the most important interventions and characteristics associated with the U5M, Bayesian geostatistical variable selection was used, adopting a stochastic search approach. The selection consisted of introducing a binary indicator parameter for each of socio-demographic, IRS and land cover variables with values defining the covariate-specific inclusion probability in the model. We assumed that the indicator arises from a Bernoulli prior distribution with probability defining the variable-specific inclusion probability in the model. We have chosen a spike and slab prior for the regression coefficients, which is a mixture of normals with mixing proportion equal to the inclusion probability. The spike component shrinks the regression coefficient to zero when the variable is excluded and the slab assumes a non-informative normal prior distribution when the variable has high inclusion probability (i.e., ≥ 50%). Environmental and climatic indicators (LST, NDVI, distance to permanent bodies and rainfall) were included or excluded in the model in a linear or categorical form. We introduced indicators with a multinomial prior distribution with three parameters corresponding to the probabilities of exclusion of a variable, inclusion in linear or categorical form. ITN coverage indicators were highly correlated with more than 85%. Hence, only one (or none) ITN indicator among those measuring ownership and one (or none) ITN indicator among those defining use was selected. The ITN indicator with the highest probability of inclusion in each category was included in the final model. Health intervention indicators were standardized and a separate model adjusting for possible confounders was fitted for each selected intervention.

Maps were generated using ArcGIS version 10.5 (ESRI; Redlands, CA, USA). Descriptive data analysis was carried out in STATA version 14.0 (Stata Corporation; College Station, TX, USA). Bayesian variable selection and model fit were implemented in OpenBUGS 3.2.3 (Imperial College and Medical Research Council; London, UK). The effects of health interventions on U5M were summarized by posterior medians of their hazard rate ratios (HRR) and the corresponding 95% Bayesian credible intervals (BCI). An estimate is considered statistically significant if its 95% BCI excludes one. Details on the Bayesian geostatistical methods are provided in the Additional file 1.

## Results

Table 3 provides a summary of the U5M estimates and the coverage of health interventions at regional and country levels.

**Table 3:** Coverage of interventions (%) and U5MR at the regional and country levels, Uganda DHS 2006 and 2011

Interventions	Central1	Central2	Kampala	East-Central	Mid-Eastern	North-East	Mid-North	West-Nile	Mid-Western	South-West	Country
Malaria											
%H_IRS	1	3	5	1	2	2	49	1	0	1	7
%H_1ITN	47	42	54	36	58	67	65	67	51	51	54
%H_1ITN2	24	21	37	11	19	27	23	25	21	20	22
%P_ITNA	37	33	47	23	38	47	45	47	38	37	39
%P_ITNS	27	24	40	19	33	45	34	40	29	26	31
%P_ITN5	47	43	62	36	50	58	53	60	50	45	38
%P_ITNU	27	24	40	19	33	45	35	41	28	26	31
WASH											
Improved water	45	69	90	78	83	85	76	71	58	45	69
Improved sanitation	25	25	17	15	9	3	5	4	9	7	14
P_wsoap	45	27	42	12	9	2	10	5	32	16	27
Reproductive health											
Family planning	35	35	48	28	23	8	17	14	27	28	27
ANC provider	89	94	97	92	95	97	96	98	95	95	95
4+ ANC visits	52	49	64	44	39	56	48	62	46	45	48
IPTp	18	19	23	15	26	29	19	18	27	24	23
Skilled delivery	57	60	91	61	47	31	42	47	44	37	50
Postnatal care	11	8	29	8	14	19	13	11	9	1	11
Breastfeeding											
Within 1 h	42	52	56	55	41	70	40	38	55	45	48
Exclusive	56	64	65	53	58	52	58	57	56	48	52
Vaccinations											
Tetanus toxoid	71	80	80	80	81	93	82	85	79	80	80
BCG	81	92	93	92	97	100	95	98	93	86	93
DPT	59	63	71	57	70	90	70	72	74	70	68
Polio	51	57	64	54	62	65	58	61	69	71	61
Measles	67	69	77	65	70	91	76	71	79	69	72
Micronutrients											
VitaminA_sup	30	36	41	56	50	74	54	42	55	37	47
Iron_sup	4	4	3	5	8	12	12	9	5	3	7
Iodized salt	99	98	100	98	99	100	99	96	94	96	98
Treatments											
Antibiotics	55	49	72	33	46	30	43	54	58	42	47
ORS or RHF	44	52	48	49	44	77	53	46	37	28	46
Zinc	1	2	2	2	1	1	2	3	3	0	2
ACT	39	34	42	34	31	81	43	40	37	30	36
Deworming	47	45	58	40	48	65	44	38	51	41	46
Mortality rates											
U5MR	83	79	56	104	80	152	76	100	95	99	90

The overall U5M was 90 deaths per 1000 live births. There were large regional variations in mortality rates with the lowest (56 deaths per 1000 live births) in Kampala and the highest (152 deaths per 1000 live births) in the North-East. The discrepancies in U5M across regions suggest that mortality rates may be influenced by region-specific factors.

IRS is the malaria intervention with the lowest coverage (7%). Among the WASH practices, the percentage of households having improved sanitation facilities was lowest (14%). Postnatal care was the least implemented reproductive health intervention with 11% of the newborns receiving the intervention. Among vaccinations, BCG had the highest coverage (93%). Almost all children (98%) lived in households that use iodized salt. Iron supplementation coverage was the lowest micronutrient intake nationally (7%) whereas zinc was the least implemented treatment (2%).

Table 4 presents results from the Bayesian geostatistical variable selection. Variables selected with 50% or higher inclusion probabilities were incorporated into the final model (e.g., improved source of drinking water and improved sanitation facilities from the WASH practices interventions).

**Table 4:** Posterior inclusion probabilities of interventions, socio-economic, demographic and environmental/climatic factors

Variable	Inclusion probability (%)
Malaria	
ITN access	
None	8.4
%H_1ITN	27.2
%H_1ITN2	4.0
%P_ITNA	60.4^a^
ITN use	
None	3.1
%P_ITNS	19.1
%P_ITN5	12.3
%P_ITNU	65.5^a^
WASH	
Improved water	66.6^a^
Improved sanitation	52.2^a^
P_wsoap	23.8
Reproductive health	
Family planning	69.0^a^
4+ ANC visits	25.0
IPTp	60.8^a^
Skilled delivery	70.0^a^
Postnatal care	76.4^a^
Breastfeeding	
Within 1 h	73.8^a^
Exclusive	100.0^a^
Vaccinations	
BCG	22.0
DPT	83.0^a^
Polio	14.8
Measles	83.4^a^
Micronutrients	
VitaminA_sup	60.0^a^
Iron_sup	38.6
Treatments	
Antibiotics	55.0^a^
ORS or RHF	57.0^a^
ACTs	61.0^a^
Deworming	81.0^a^
Socio-economic and demographic	
Child	
Sex	73.2^a^
Maternal	
Age at birth	100.0^a^
Number of children	100.0^a^
Education level	70.2^a^
Residence (urban vs rural)	59.6^a^
Working status	34.0
Household	
Age of head	0.0
Wealth index	81.2^a^
Environmental/Climatic factors	
Land cover	33.0
LST day: None	73.0
LST day continuous	27.0
LST day categorical	0.0
LST night: None	90.6
LST night continuous	8.1
LST night categorical	1.3
NDVI: None	41.2
NDVI continuous	58.8^a^
NDVI categorical	0.0
Rainfall: None	80.3
Rainfall continuous	12.2
Rainfall categorical	8.5
d_water: None	100.0
d_water continuous	0.0
d_water categorical	0.0

^a^Selected variables with > = 50% inclusion probability: *LST* land surface temperature, *NDVI* normalized difference vegetation index, *d_water* distance to permanent water bodies

Results presented in Table 5 show that at the national level all interventions except family planning were associated with a lower risk of U5M, with ACT associated with a highest reduction (HRR = 0.60; 95% BCI: 0.11, 0.79).

**Table 5:** Posterior estimates of interventions’ effects at national and sub-national scale on U5MR adjusted for confounders

**Geographical scale**	**Malaria**		**WASH**		**Reproductive health**			
	**%P_ITNA**	**%P_ITNU**	**Improved water**	**Improved sanitation**	**Family planning**	**IPT**	**Skilled delivery**	**Postnatal care**
	**HRR (95% BCI)**	**HRR (95% BCI)**	**HRR (95% BCI)**	**HRR (95% BCI)**	**HRR (95% BCI)**	**HRR (95% BCI)**	**HRR (95% BCI)**	**HRR (95% BCI)**
**National**	^a^0.75 (0.63, 0.84)*	0.88 (0.83, 0.94)*	0.76 (0.70, 0.81)*	0.86 (0.74, 0.91)*	1.04 (0.92, 1.10)	0.74 (0.67, 0.97)*	0.84 (0.78, 0.90)*	0.80 (0.74, 0.89)*
**Region**								
Central 1	0.54 (0.35, 0.81)*	0.56 (0.51, 0.63)*	0.99 (0.91, 1.14)	1.13 (0.89, 1.36)	1.29 (0.82, 2.08)	0.73 (0.51, 0.98)*	1.09 (0.84, 2.26)	0.82 (0.61, 0.96)*
Central 2	0.63 (0.51, 0.85)*	0.94 (0.85, 1.07)	0.87 (0.67, 0.98)*	0.99 (0.79, 1.23)	0.70 (0.46, 0.85)*	0.82 (0.57, 1.72)	1.06 (0.80, 1.22)	0.73 (0.63, 0.97)*
East-Central	0.98 (0.73, 1.33)	1.02 (0.90, 1.13)	0.82 (0.70, 0.91)*	1.25 (0.76, 1.90)	1.20 (0.85, 1.98)	0.83 (0.58, 1.12)	1.25 (0.94, 1.62)	0.76 (0.65, 0.95)*
Kampala	0.70 (0.51, 0.87)*	0.81 (0.73, 0.97)*	0.77 (0.69, 0.92)*	0.89 (0.66, 1.17)	0.85 (0.73, 0.97)*	0.25 (0.14, 0.44)*	0.81 (0.72,0.92)*	0.58 (0.50, 0.78)*
Mid-Eastern	1.04 (0.45, 2.36)	0.87 (0.67, 1.31)	0.71 (0.59, 0.77)*	0.84 (0.65, 1.11)	1.29 (0.93, 1.88)	0.35 (0.23, 0.73)*	1.15 (0.72, 1.58)	0.92 (0.68, 1.17)
Mid-North	0.82 (0.54, 1.15)	0.77 (0.62, 0.87)*	0.50 (0.38, 0.57)*	1.02 (0.76, 1.29)	1.03 (0.79, 1.28)	1.12 (0.64, 1.50)	0.81 (0.61, 0.95)*	1.05 (0.58, 1.29)
Mid-Western	0.46 (0.21, 0.84)*	1.02 (0.91, 1.35)	1.04 (0.81, 1.41)	1.01 (0.77, 1.17)	1.00 (0.48, 1.56)	0.60 (0.46, 0.85)*	0.49 (0.40, 0.68)*	0.70 (0.45, 0.84)*
North-East	1.49 (0.96, 2.14)	1.23 (0.99, 1.39)	1.09 (0.92, 1.42)	0.50 (0.39, 0.66)*	0.77 (0.62, 0.89)*	0.83 (0.56, 1.28)	0.77 (0.68, 0.86)*	1.06 (0.87, 1.35)
South-West	0.34 (0.22, 0.63)*	0.75 (0.64, 0.87)*	0.89 (0.80, 1.02)	0.51 (0.28, 0.67)*	1.38 (0.83, 2.08)	0.82 (0.46, 1.32)	0.68 (0.52, 0.82)*	0.71 (0.56, 1.21)
West-Nile	1.24 (0.93, 1.87)	0.95 (0.83, 1.05)	0.30 (0.23, 0.48)*	0.69 (0.53, 0.87)*	0.81 (0.65, 0.96)*	0.68 (0.45, 0.93)*	0.69 (0.23, 0.98)*	0.82 (0.60, 1.24)
**Spatial parameters**	**Median (95% BCI)**	**Median (95% BCI)**	**Median (95% BCI)**	**Median (95% BCI)**	**Median (95% BCI)**	**Median (95% BCI)**	**Median (95% BCI)**	**Median (95% BCI)**
Spatially varying^b^	0.65 (0.43, 0.90)	0.49 (0.43, 0.54)	0.59 (0.45, 0.64)	0.63 (0.57, 1.11)	0.59 (0.38, 0.84)	0.58 (0.48, 1.03)	0.54 (0.35, 0.84)	0.51 (0.37, 0.47)
Spatial process	0.29 (0.16, 0.34)	0.30 (0.21, 0.36)	0.21 (0.15, 0.25)	0.23 (0.19, 0.27)	0.27 (0.19, 0.31)	0.15 (0.11, 0.20)	0.31 (0.19, 0.41)	0.29 (0.16, 0.34)
Range (km)^c^	3.33 (0.55, 5.83)	1.06 (0.32, 4.21)	0.46 (0.35, 3.83)	0.56 (0.36, 1.84)	0.81 (0.31, 1.38)	0.37 (0.32, 3.49)	0.71 (0.32, 3.18)	3.33 (0.53, 5.83)
**Other parameters**								
Shape parameter^d^	0.44 (0.41, 0.48)	0.41 (0.36, 0.49)	0.35 (0.32, 0.38)	0.38 (0.28, 0.42)	0.30 (0.27, 0.36)	0.37 (0.31, 0.43)	0.44 (0.39, 0.47)	0.46 (0.42, 0.53)
Confounders; socio-demographic and environmental/climatic factors, *significant and protective, HRR; Hazard rate ratio, %P_ITNA; Percentage of population with access to an ITN within their household, %P_ITNU; Percentage of existing ITNs used by the population in a household the previous night of the survey, a Intervention coverage was modeled on a standardized scale; therefore results are interpreted as associations. The coverage of P_ITNA was associated with a reduction in the mortality rate of 0.25; (HR = 0.75; 95% BCI: 0.63, 0.84), bIndicates the degree of variation in associations between interventions and mortality in the country, cMeasures distance after which spatial correlation between mortality at clusters becomes negligible and dDescribes the trend in the baseline mortality hazard over time								
**Geographical scale**	**Breastfeeding**	**Vaccinations**		**Micronutrients**	**Treatments**			
	**Within 1 h**	**DPT**	**Measles**	**VitaminA_sup**	**Deworming**	**ORS or RHF**	**ACT**	
	**HRR (95% BCI)**	**HRR (95% BCI)**	**HRR (95% BCI)**	**HRR (95% BCI)**	**HRR (95% BCI)**	**HRR (95% BCI)**	**HRR (95% BCI)**	
**National**	0.70 (0.51, 0.86)*	0.85 (0.72, 0.97)*	0.82 (0.69, 0.89)*	0.88 (0.78, 0.97)*	0.89 (0.84, 0.94)*	0.86 (0.78, 0.92)*	0.60 (0.11, 0.79)*	
**Region**								
Central 1	0.55 (0.51, 0.92)*	0.60 (0.44, 0.81)*	0.63 (0.39, 0.88)*	0.74 (0.58, 0.97)*	0.76 (0.37, 1.69)	0.30 (0.21, 0.74)*	0.41 (0.11, 0.94)*	
Central 2	0.63 (0.54, 1.43)	0.95 (0.75, 1,23)	0.79 (0.48, 1.47)	0.86 (0.64, 1.15)	1.25 (0.78, 1.89)	1.27 (0.79, 1.42)	0.56 (0.31, 1.10)	
East-Central	0.88 (0.73, 1.72)	0.74 (0.55, 1.07)	0.97 (0.61, 1.36)	1.09 (0.81, 1.53)	1.08 (0.93, 1.24)	1.35 (0.89, 2.72)	0.84 (0.54, 1.52)	
Kampala	0.53 (0.39, 0.91)*	0.82 (0.70, 1.25)	0.63 (0.29, 0.94)*	0.77 (0.56, 1.17)	0.72 (0.60, 0.89)*	0.34 (0.18, 0.72)*	0.32 (0.09, 0.80)*	
Mid-Eastern	0.93 (0.83, 1.90)	0.67 (0.47, 0.93)*	1.38 (0.85, 2.01)	1.12 (0.64, 2.20)	0.50 (0.23, 1.42)	2.30 (0.79, 2.72)	1.33 (0.78, 1.78)	
Mid-North	0.91 (0.83, 1.46)	0.69 (0.38, 0.85)*	0.71 (0.47, 1.31)	1.05 (0.73, 1.63)	1.16 (0.79, 1.88)	1.05 (0.46, 1.34)	0.77 (0.40, 1.66)	
Mid-Western	0.71 (0.68, 1.27)	1.12 (0.76, 1.42)	0.88 (0.55, 1.21)	1.12 (0.78, 1.63)	0.93 (0.73, 1.14)	0.92 (0.68, 1.21)	0.49 (0.22, 0.79)*	
North-East	0.98 (0.91, 1.69)	0.96 (0.73, 1.32)	1.29 (0.93, 1.60)	1.31 (0.93, 1.65)	1.18 (0.99, 1.30)	1.10 (0.86, 1.42)	0.89 (0.72, 1.44)	
South-West	0.56 (0.42, 0.82)*	1.61 (0.63, 2.25)	0.64 (0.38, 0.92)*	0.63 (0.43, 0.81)*	0.74 (0.62, 0.92)*	0.75 (0.54, 0.85)*	0.40 (0.23, 0.61)*	
West-Nile	0.63 (0.55, 0.98)*	0.86 (0.62, 0.98)*	0.65 (0.29, 1.23)	0.49 (0.34, 0.67)*	0.85 (0.73, 0.98)*	0.75 (0.28, 1.75)	0.66 (0.29, 3.81)	
**Spatial parameters**	**Median (95% BCI)**	**Median (95% BCI)**	**Median (95% BCI)**	**Median (95% BCI)**	**Median (95% BCI)**	**Median (95% BCI)**	**Median (95% BCI)**	
Spatially varying^b^ (Ω_*d*_)	0.50 (0.33, 0.72)	0.62 (0.43. 0.80)	0.52 (0.34, 1.12)	0.56 (0.36, 0.75)	0.58 (0.36, 0.98)	0.79 (0.52, 0.92)	0.66 (0.42, 1.56)	
Spatial process (*σ*^2^)	0.13 (0.09, 0.22)	0.27 (0.19, 0.36)	0.23 (0.18, 0.30)	0.15 (0.12, 0.22)	0.27 (0.22, 0.34)	0.20 (0.18, 0.24)	0.36 (0.27, 0.51)	
Range (km)^c^ (*R*)	0.71 (0.31, 3.78)	0.45 (0.31, 0.48)	0.70 (0.33, 1.51)	0.48 (0.31, 1.82)	0.57 (0.35, 1.56)	0.44 (0.35, 1.68)	0.60 (0.32, 2.01)	
**Other parameters**								
Shape parameter^d^ (*r*)	0.37 (0.34, 0.41)	0.41 (0.33, 0.45)	0.40 (0.38, 0.44)	0.42 (0.38, 0.46)	0.35 (0.31, 0.42)	0.37 (0.32, 0.43)	0.44 (0.40, 0.53)	
Confounders; socio-economic, demographic and environmental/climatic factors,*significant and protective, HRR; Hazard rate ratio, ORS or RHF; Percentage of children with diarrhoea given fluid from ORS sachets or recommended home fluids, ACT; Percentage of children with fever during the 2 weeks prior to the survey and took artemisinin-combination therapy, bIndicates the degree of variation in associations between interventions and mortality in the country, cMeasures distance after which spatial correlation between mortality at clusters becomes negligible and dDescribes the trend in the baseline mortality hazard rate over time								

**Fig. 1:**
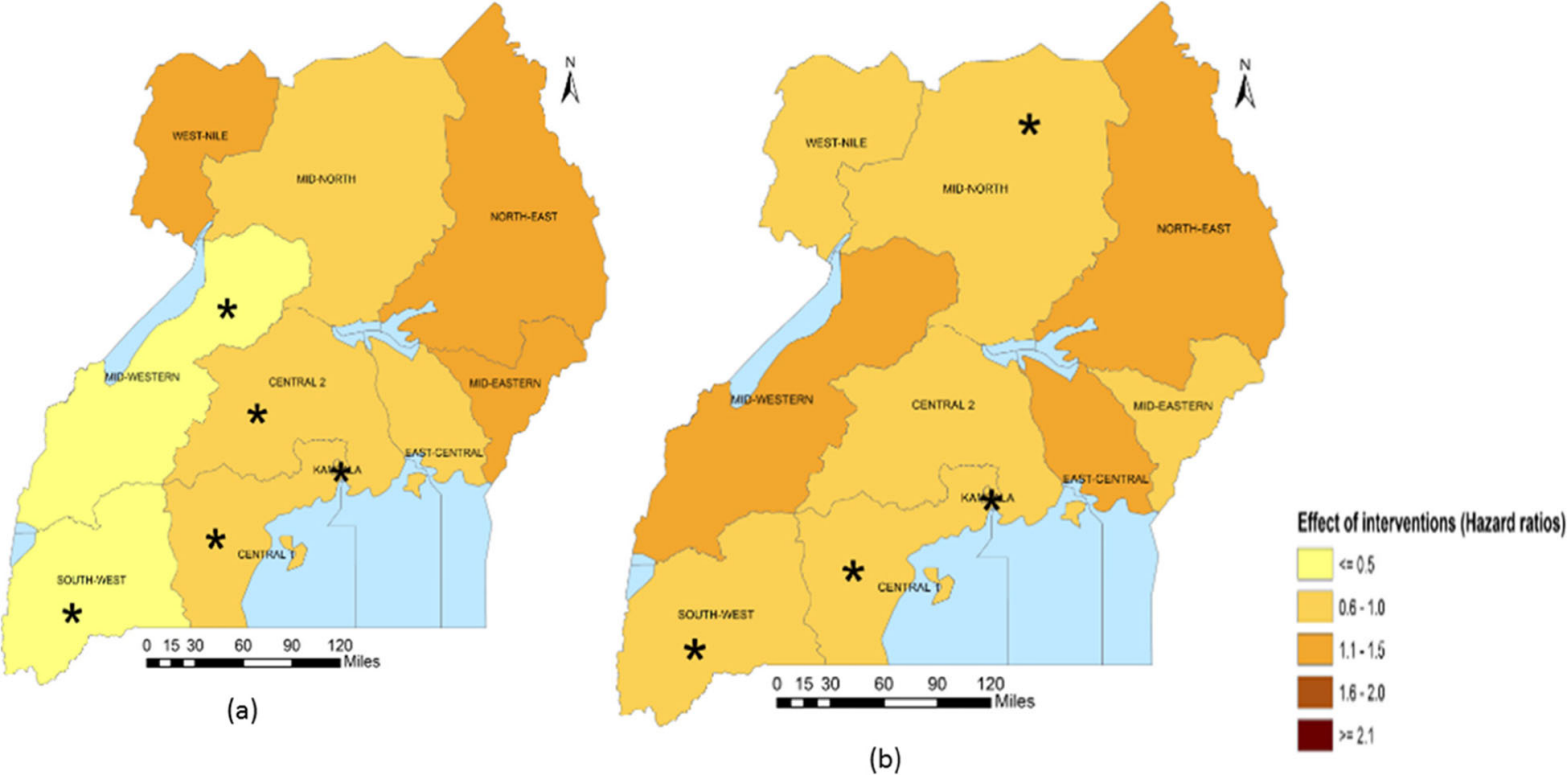
Geographical distribution of the associations (Hazard rate ratios) of malaria interventions with under-five mortality; (*statistically significant association of interventions and protective against mortality); **a** Percentage of population with access to an ITN within their household, **b** Percentage of existing ITNs used by the population in a household the previous night of the survey

**Fig. 2:**
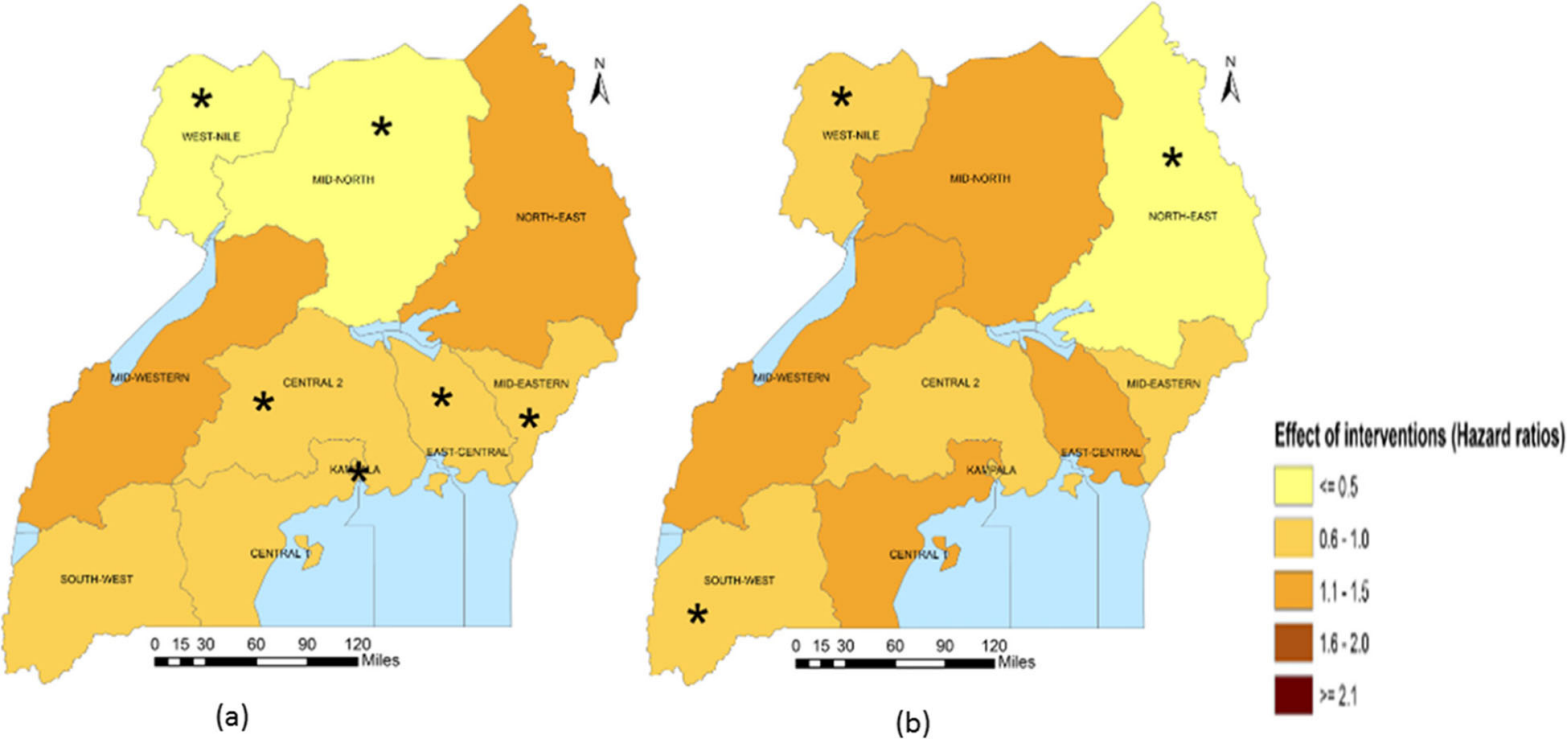
Geographical distribution of the associations (Hazard rate ratios) of water, sanitation and hygiene practices with under-five mortality; (*statistically significant effect of interventions and protective against mortality); **a** Percentage of households with improved source of drinking water, **b** Percentage of households using improved sanitation facilities

**Fig. 3:**
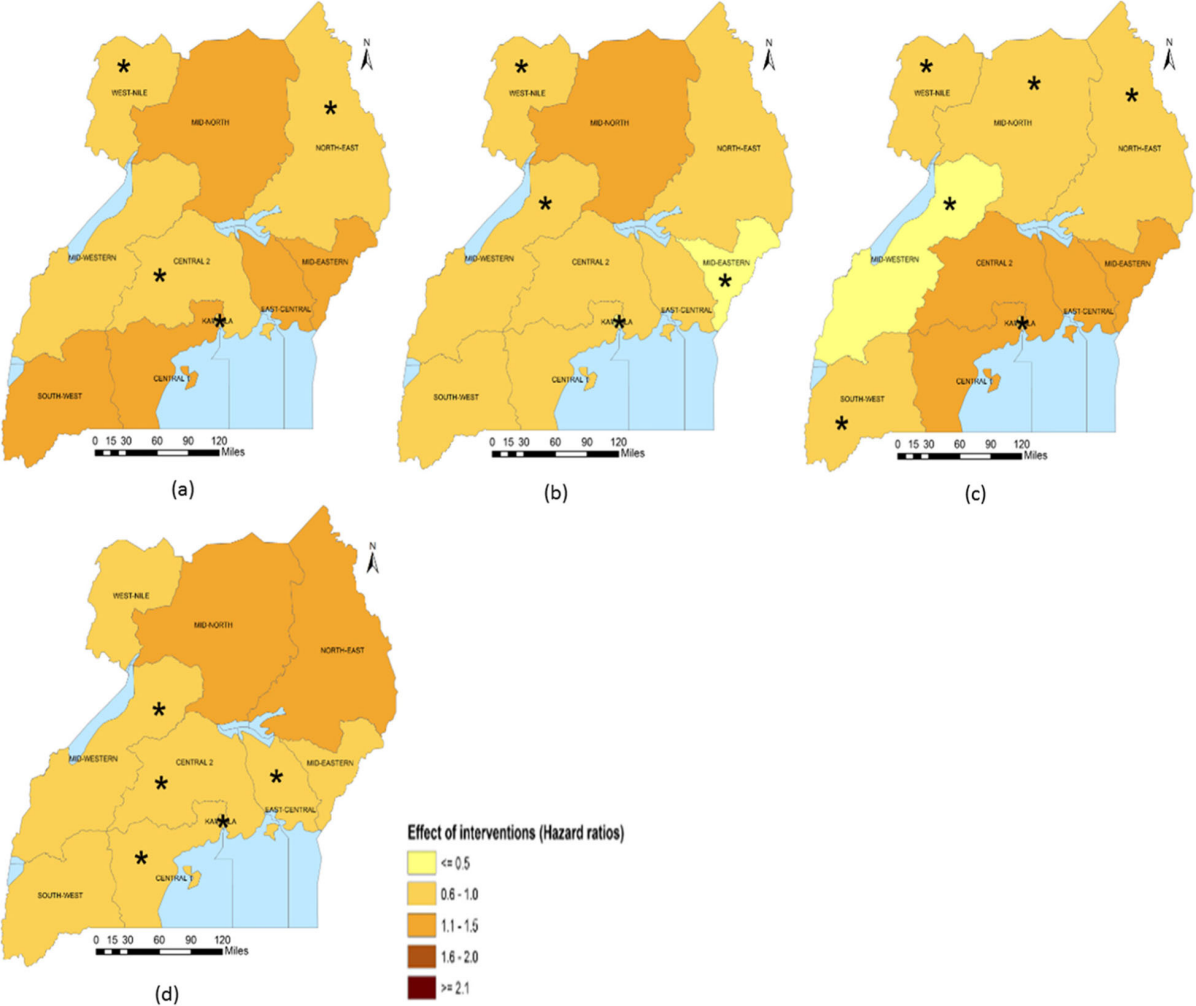
Geographical distribution of the associations (Hazard rate ratios) of reproductive health interventions with under-five mortality; (*statistically significant association of interventions and protective against mortality); **a** Percentage of married women using any family planning method, **b** Percentage of women who received intermittent preventive treatment for malaria during pregnancy, **c** Percentage of births that took place with the assistance of a skilled provider, **d** Percentage of newborns receiving first postnatal checkup from a skilled provider within 2 days after delivery

**Fig. 4:**
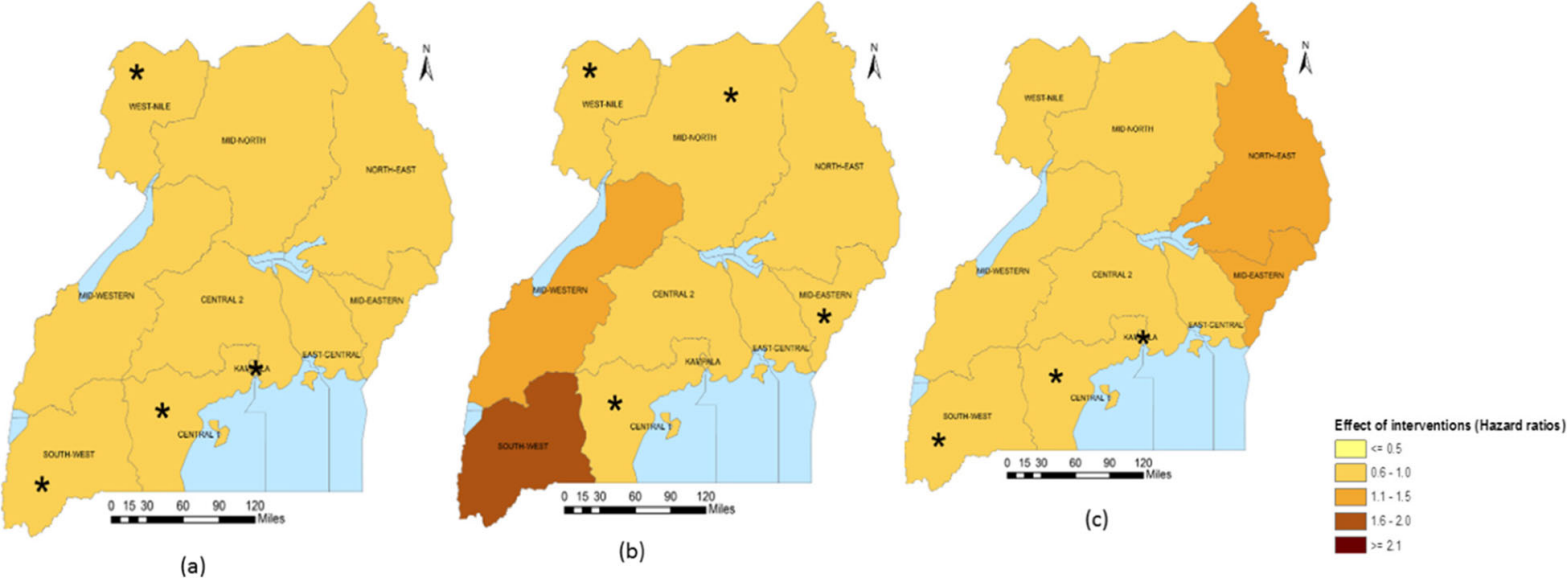
Geographical distribution of the associations (Hazard rate ratios) of breastfeeding and vaccinations with under-five mortality; (*statistically significant association of interventions and protective against mortality); **a** Percentage of infants who started breastfeeding within 1 h of birth, **b** Percentage of children with complete vaccination of DPT, **c** Percentage of children vaccinated against measles

**Fig. 5:**
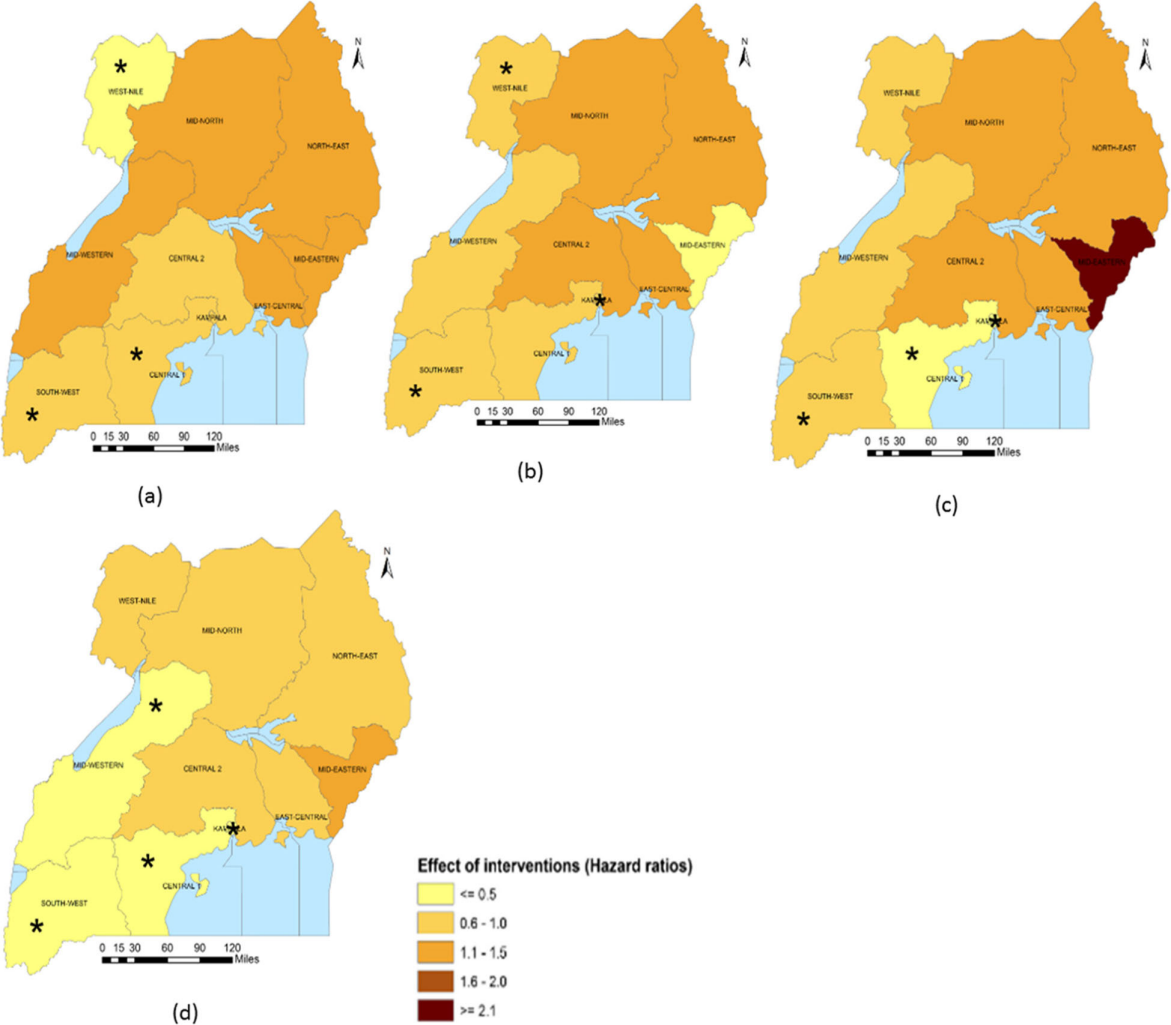
Geographical distribution of the associations (Hazard rate ratios) of micronutrients intake and treatments with under-five mortality; (*statistically significant association of interventions and protective against mortality); **a** Percentage of children receiving vitamin A supplements in the past 6 months, **b** Percentage of children given deworming medication in the past 6 months, **c** Percentage of children with diarrhoea given fluid from oral rehydration solution sachets or recommended home fluids, **d** Percentage of children with fever during the 2 weeks prior to the survey and took artemisinin-combination therapy

Sub-national analysis (Table 5) indicates that in Central 2, Mid-Western and South-West regions, the largest reduction in the U5M burden was associated with ITN access. The intervention also had a large association on U5M in Central 1 and Kampala. Improved source of drinking water was associated with most U5M decrease in Mid-North and West-Nile. Improved source of drinking water was, in addition, associated with U5M in Central 2, East-Central, Kampala and Mid-Eastern. Improved sanitation facilities were associated with the highest decline in U5M in the North-East. The coverage of improved sanitation facilities also had an important association with mortality in South-West and West-Nile. In Kampala and Mid-Eastern, IPTp, had the largest association with U5M. The relation between IPTp and mortality was statistically important in Central 1, Mid-Western and West-Nile. In Central 1 and East-Central, ORS or RHF and postnatal care were respectively associated with the highest decreases in U5M. The coverage of postnatal had an important association on U5M in Central 1, Central 2, Kampala and Mid-Western. ORS or RHF was associated with lower mortality hazards in Kampala and South-West. Family planning had an important association on mortality in Central 2, Kampala, North-East and West-Nile. Figures 1, 2, 3, 4 and 5 summarize graphically the spatially varying effects of all interventions on U5M.

Table 6 show that socio-demographic, environmental and climatic factors were important determinants of U5M. For instance, children born to mothers residing in urban areas had lower hazards of mortality relative to those in rural areas. Environmental and climatic factors were associated with lower hazards. Children living in areas with a higher NDVI showed increased survival times.

**Table 6:** Posterior estimates for effects of socio-demographic and environmental/climatic factors adjusted for in each intervention model

**Variable**	**Malaria**		**WASH**		**Reproductive health**			
	**%P_ITNA**	**%P_ITNU**	**Improved water**	**Improved sanitation**	**Family planning**	**IPT**	**Skilled delivery**	**Postnatal care**
	**HRR (95% BCI)**	**HRR (95% BCI)**	**HRR (95% BCI)**	**HRR (95% BCI)**	**HRR (95% BCI)**	**HRR (95% BCI)**	**HRR (95% BCI)**	**HRR (95% BCI)**
**Socio-demographic factors**								
**Child**								
*Sex*								
Male	1.0	1.0	1.0	1.0	1.0	1.0	1.0	1.0
Female	0.82(0.76,0.90)^a^	0.89(0.75,0.96)^a^	0.84(0.73,0.98)^a^	0.80(0.78,0.94)^a^	0.86(0.73,0.93)^a^	0.78(0.73,0.86)^a^	0.85(0.72,0.91)^a^	0.81(0.78,0.98)^a^
**Maternal**								
*Age at birth*								
15–24	1.0	1.0	1.0	1.0	1.0	1.0	1.0	1.0
25–29	0.60(0.51,0.72)^a^	0.61(0.55,0.69)^a^	0.62(0.48,0.71)^a^	0.59(0.52,0.66)^a^	0.62(0.46,0.75)^a^	0.69(0.54,0.73)^a^	0.63(0.49,0.80)^a^	0.67(0.60,0.73)^a^
30–34	0.51(0.42,0.58)^a^	0.38(0.35,0.46)^a^	0.38(0.31,0.45)^a^	0.44(0.37,0.51)^a^	0.43(0.30,0.59)^a^	0.49(0.42,0.52)^a^	0.45(0.32,0.62)^a^	0.52(0.47,0.57)^a^
35–49	0.62(0.58,0.69)^a^	0.50(0.41,0.59)^a^	0.50(0.44,0.56)^a^	0.52(0.44,0.58)^a^	0.48(0.33,0.71)^a^	0.61(0.48,0.71)^a^	0.50(0.34,0.73)^a^	0.71(0.62,0.77)^a^
*#Children born*	1.44(1.36,1.57)^a^	1.53(1.48,1.62)^a^	1.52(1.43,1.62)^a^	1.53(1.36,1.56)^a^	1.53(1.29,1.73)^a^	1.45(1.32,1.59)^a^	1.49(1.30,1.71)^a^	1.38(1.30,1.49)^a^
*Residence*								
Rural	1.0	1.0	1.0	1.0	1.0	1.0	1.0	1.0
Urban	1.10(0.85,1.55)	1.03(0.86,1.25)	1.16(0.99,1.36)	1.11(0.92,1.26)	1.18(0.82,1.64)	1.20(1.01,1.38)	1.13(0.85,1.49)	1.00(0.96,1.12)
*Education level*								
None	1.0	1.0	1.0	1.0	1.0	1.0	1.0	1.0
Primary	1.14(0.89,1.45)	1.14(0.89,1.45)	1.12(0.88,1.42)	1.13(0.89,1.43)	1.16(0.91,1.49)	1.13(0.89,1.44)	1.19(0.93,1.51)	1.11(0.87,1.41)
Secondary+	1.22(0.87,1.70)	1.22(0.87,1.71)	1.22(0.87,1.70)	1.21(0.86,1.69)	1.26(0.90,1.78)	1.21(0.86,1.69)	1.30(0.93,1.83)	1.20(0.86,1.68)
**Household**								
*Wealth index*	0.90(0.82,0.98)^a^	0.91(0.81,0.98)^a^	0.86(0.81,0.92)^a^	0.86(0.82,0.96)^a^	0.90(0.78,0.97)^a^	0.88(0.78,0.94)^a^	0.95(0.84,1.07)	0.84(0.81,0.90)^a^
**Environmental/climatic factors**								
*NDVI*	0.85(0.80,0.96)^a^	0.98(0.89,1.02)	0.83(0.77,0.92)^a^	0.95(0.92,1.02)	1.00(0.85,1.17)	0.95(0.90,0.98)^a^	0.95(0.86,1.05)	0.89(0.84,0.96)^a^
**Variable**	**Breastfeeding**	**Vaccinations**		**Micronutrients**	**Treatments**			
	**Within 1 h**	**DPT**	**Measles**	**VitaminA_sup**	**Deworming**	**ORS or RHF**	**ACT**	
	**HRR (95% BCI)**	**HRR (95% BCI)**	**HRR (95% BCI)**	**HRR (95% BCI)**	**HRR (95% BCI)**	**HRR (95% BCI)**	**HRR (95% BCI)**	
**Socio-demographic factors**								
**Child**								
*Sex*								
Male	1.0	1.0	1.0	1.0	1.0	1.0	1.0	
Female	0.86(0.72,0.98)^a^	0.91(0.87,0.96)^a^	0.79(0.65,0.92)^a^	0.87(0.69,0.98)^a^	0.84(0.77,0.91)^a^	0.85(0.62,0.99)^a^	0.78(0.69,0.85)^a^	
**Maternal**								
*Age at birth*								
15–24	1.0	1.0	1.0	1.0	1.0	1.0	1.0	
25–29	0.69(0.51,0.72)^a^	0.62(0.51,0.69)^a^	0.69(0.58,0.82)^a^	0.63(0.55,0.68)^a^	0.59(0.53,0.75)^a^	0.58(0.49,0.67)^a^	0.61(0.53,0.71)^a^	
30–34	0.49(0.42,0.58)^a^	0.48(0.38,0.54)^a^	0.45(0.39,0.57)^a^	0.41(0.35,0.54)^a^	0.41(0.34,0.54)^a^	0.41(0.32,0.51)^a^	0.37(0.31,0.43)^a^	
35–49	0.62(0.47,0.79)^a^	0.47(0.36,0.57)^a^	0.57(0.49,0.64)^a^	0.48(0.34,0.55)^a^	0.40(0.36,0.59)^a^	0.45(0.33,0.56)^a^	0.42(0.36,0.55)^a^	
*#Children born*	1.46(1.28,1.51)^a^	1.59(1.48,1.67)^a^	1.39(1.30,1.45)^a^	1.54(1.34,1.78)^a^	1.69(1.57,1.72)^a^	1.59(1.44,1.79)^a^	1.63(1.56,1.70)^a^	
*Residence*								
Rural	1.0	1.0	1.0	1.0	1.0	1.0	1.0	
Urban	1.06(0.87,1.45)	0.90(0.86,0.97)^a^	1.04(0.80,1.26)	1.23(0.89,1.47)	1.03(0.95,1.12)	1.18(0.80,1.62)	0.98(0.85,1.11)	
*Education level*								
None	1.0	1.0	1.0	1.0	1.0	1.0	1.0	
Primary	1.25(0.91,1.47)	1.18(0.92,1.51)	1.18(0.92,1.52)	1.35(0.99,163)	1.15(0.90,1.47)	1.16(0.91,1.49)	1.29(0.93,1.67)	
Secondary+	1.36(0.98,1.70)	1.27(0.90,1.79)	1.27(0.90,1.79)	1.27(0.94,1.67)	1.23(0.87,1.72)	1.24(0.88,1.73)	1.38(0.98,1.96)	
**Household**								
*Wealth index*	0.91(0.80,0.98)^a^	0.93(0.81,0.98)^a^	0.93(0.87,0.96)^a^	0.87(0.81,0.98)^a^	0.92(0.91,0.98)^a^	0.88(0.82,0.97)^a^	1.03(0.92,1.21)	
**Climatic/Environmental factors**								
*NDVI*	0.97(0.90,1.11)	1.06(0.99,1.15)	1.02(0.89,1.09)	0.98(0.88,1.10)	1.02(0.95,1.11)	1.01(0.86,1.13)	0.97(0.87,1.10)	

^a^Statistically significant, socio-demographic; socio-economic and demographic factors

## Discussion

We quantified the associations of health interventions on all-cause U5M at national and sub-national scales in Uganda. The analysis took into account confounding effects of socio-demographic and environmental and climatic factors, which have been shown to be significantly associated with mortality [1–7]. We found strong geographical variations in the effects of health interventions on all-cause U5M across Uganda.

Findings at the national level indicated that ACT, initiation of breast feeding within 1 h of birth, IPTp, ITN access and improved source of drinking water were the health interventions associated with a highest reduction in U5M. However, these interventions were poorly implemented in the country with coverage below 50% yet the prevalences of diseases targeted by these interventions are high [8]. For example, malaria prevalence was at least 40% nationally and in 80% of the regions [9]. Other interventions, which were associated with a significant reduction in mortality U5M include ITN use, improved sanitation facilities, skilled delivery, postnatal care, complete DPT and measles vaccination, vitamin A supplementation, deworming in the past 6 months and ORS or RHF. These variables are among the essential health interventions that have been associated with a decrease in child mortality in a review by Lassi et al. [10]. Our findings corroborate results reported from analyses of DHS data [11] and community-based studies in Uganda [12, 13]. Similar results were reported in other settings. For instance, Masanja et al. in Tanzania [14] analysed DHS data and found that increased coverage of key child-survival interventions, such as sleeping under ITNs, vitamin A supplementation, immunisation and exclusive breastfeeding accelerated progress in reducing U5M in Tanzania. In addition, analysis of cohort studies in Burkina Faso [15], randomised control trials in Guinea Bissau [16] and systematic reviews [17, 18] reported vaccinations to be associated with declines in child mortality. Our findings showed that DPT and measles vaccination were not statistically associated with U5M in Uganda although they had high coverage. This could be related to the untimely receipt of the vaccines, which might have hindered optimal immune response to the vaccines. According to guidelines developed by the World Health Organization (WHO) [19], children are considered fully vaccinated when they have received a vaccination against tuberculosis (BCG), three doses each of the diphtheria, pertussis and tetanus (DPT) and polio vaccines and a measles vaccination by the age of 12 months. At the time of the survey, only half of the children had received all basic vaccinations by the appropriate age of 12 months [8]. Untimely vaccinations contribute to coverage figures leading to an overestimation of actual population immunity [20–22].

The varying associations between interventions and U5M across regions could be explained by external factors, which this analysis has not taken into account but influence the effect of interventions, such as the health system, which differs between regions. This suggests for further efforts beyond increasing coverage of interventions, such as an approach which improves external factors as well as intervention coverage.

Study results confirmed earlier finding that household socioeconomic status is protective against U5M. The improved socioeconomic status boosts the effect of interventions despite their low coverages. Better socioeconomic status has been shown to reduce U5M [23–26]. In Central 2, Mid-Western and South-West, in which ITN access had the largest association with mortalityU5M, over 65% of households in the three regions fall either in the middle, fourth or highest wealth quintile [8]. More than 65% percent of households in East-Central, in which postnatal care had a leading association with U5M, are in the middle, fourth or highest wealth quintile [8]. IPTp had a strongest association with mortality in Kampala and over 90% of households in this region are in the highest quintile [8]. In Central 1 where ORS or RHF had a highest association with U5M, over 65% of households either belong to the fourth or highest wealth quintile [8]. The improved socioeconomic status in these regions might have contributed to the success of the poorly implemented interventions. The North-East, in which coverage of several interventions was satisfactory (e.g., improved source of drinking water, DPT and measles vaccination, deworming, ORS or RHF and ACT), over 80% of households in this region are in the lowest wealth quintile [8]. The high poverty level in the North-East could have hindered the effectiveness of many interventions, which were adequately implemented in this region.

This work comes with a number of limitations. First, data on several health interventions for dead children could not be collected in the DHS. Hence, the intervention-mortality relation could not be estimated using interventions data at an individual level. Therefore, we created intervention coverage indicators measuring the proportion of children using an intervention at the cluster level. We linked individual U5M data to cluster intervention coverage and quantified associations between interventions and U5M while adjusting for socio-demographics and environmental/climatic factors.

Further, the analysis in this work was based on aggregations of the coverages of interventions at the cluster level, hence considering coverages of only living children. This could have resulted in a bias in associations due to the difference in the coverage of health interventions between the living and dead. This is because the association between coverage of interventions and mortality may differ between the living and dead children, and ignoring coverage of interventions for the dead children could induce selection bias in estimates of the coverage of interventions at a cluster level and also in estimates of associations between coverage of interventions and mortality [27]. Therefore, there is need to implement a correction to the observed coverage of interventions. Reniers et al. [28] proposed a correction based on the Bayes’ theorem and an initial estimate of the relative risk of mortality for each intervention that was obtained from the data. Several researchers including McGovern and Canning [27], have implemented this correction. Despite the correction, there were not significant differences between results obtained from the raw and corrected data, both in magnitude and direction. Most risk ratios were exactly the same or differed by 0.1. This may imply that applying the correction to our data may not greatly affect the conclusions drawn based on the findings of this manuscript [27].

Another limitation of the paper is that the causal effects of health interventions on U5M cannot be identified. This is because the DHS data used are cross-sectional and can only capture cross-sectional associations rather than effects that reflect causal interpretation.

DHS surveys are designed to estimate interventions at a region level. Other combinations of strata in the DHS are the rural and urban areas. Thus, regions could be separated into urban and rural portions and estimate the coverage of interventions within each area. This would increase the number of analysis units from regions to rural and urban areas. The methodology used in this study allows the estimation of the associations of interventions at higher resolution (i.e., pixel level). However, estimates at higher resolution would require a predictive model with high resolution estimates of the coverage of interventions. Hence, there is a research need to identify predictors of intervention coverage at very high resolution.

Regardless of these limitations, our analytical approach enabled estimation of the geographical variations in the effects of health interventions on U5M, which informs countries about the effects of interventions at a sub-national scale, so that appropriate interventions can be implemented and monitored over time.

## Conclusions

We demonstrated that the associations of health interventions with U5M vary across regions in Uganda and identified interventions associated with largest reductions in U5M by region. These findings can guide control programmes to implement the most appropriate interventions at a local scale, especially at regions. This may reduce within country U5M inequalities and consequently result into achieving national SDG mortality targets.

The coverage of interventions associated with the highest reduction in mortality in each region should be improved. In Central 2, Mid-Western and South-West regions, ITN access should be strengthened. There is a need to increase coverage of improved sources of drinking water in Mid-North and West-Nile, while efforts should be made on improving sanitation facilities in the North-East. IPTp coverage should be scaled up in Kampala and Mid-Eastern. ORS or RHF and postnatal care should be prioritized in Central 1 and East-Central respectively. Moreover, the Uganda government should improve the socioeconomic status of regions to enhance intervention performance and improve mortality rates.

## Supplementary information

**Additional file 1.** Bayesian geostatistical methods.

## Data Availability

The datasets supporting conclusions of this article were requested from the DHS program website (www.dhsprogram.com) and can be accessed following instructions at https://dhsprogram.com/data/Access-Instructions.cfm.

## Abbreviations

ACT: Artemisinin-based combination therapy
ANC: Antenatal care
ARIs: Acute respiratory infections
BCG: Bacillus Calmette Guerin
BCI: Bayesian credible interval
CA: California
DHS: Demographic and health survey
DPT: Diphtheria, pertussis and tetanus
HRR: Hazard rate ratio
IGBP: International Global Biosphere Programme
IPTp: Intermittent preventive treatment for pregnancy
IRS: Indoor residual spraying
ITN: Insecticide-treated net
LST: Land surface temperature
MIS: Malaria Indicator Survey
MODIS: Moderate resolution imaging spectroradiometer
NDVI: Normalized difference vegetation index
ORS: Recommended home fluids
RHF: Oral rehydration solution
SDG: Sustainable development goal
SRTM: Shuttle radar topographic mission
TX: Texas
U5M: Under-five mortality
UBOS: Uganda Bureau of Statistics
UK: United Kingdom
USA: United States of America
USAID: United States Agency for International Development
USGSS: United States Geological Survey-Earth Resources Observation Systems
WASH: Water, sanitation and hygiene
