# Supplementary material for: Geographical variations of the associations between health interventions and all-cause under-five mortality in Uganda
Source: BMC Public Health. 2019 Oct 22;19:1330. doi: 10.1186/s12889-019-7636-x (PMC6805502; doi:10.1186/s12889-019-7636-x)
Supplement: Supplementary file 1 — Additional file 1. Bayesian geostatistical methods. [file 12889_2019_7636_MOESM1_ESM.docx]

**Geostatistical proportional hazards model with spatially varying covariates**

A Bayesian geostatistical proportional hazards model [35] was fitted to assess the association between under-five mortality and health interventions, and to identify interventions which contribute most to mortality at the sub-national level. Let $s=\{{s_{1},s_{2}, \ldots, s_{m}\}}^{t},s_{i}\in D\subset R^{2}$ be the set of locations at which mortality data are observed, $t_{j}(s_{i})$ be the observed number of months lived or the censoring time for child $j$ at location $s_{i}$, $\boldsymbol{X}_{\boldsymbol{j}}(s_{i})$ be the vector of socio-demographic and climatic factors and $Z_{d}(s_{i})$ be the coverage of intervention $d$ at location $s_{i}$.We modeled the hazard of death by the equation, $h\left( t_{j}(s_{i}) \right)=h_{0}\left( t_{j}(s_{i}) \right)\exp\left( \boldsymbol{\beta}^{T}\boldsymbol{X}_{\boldsymbol{j}}(s_{i})+\sum_{d=1}^{D} \left( b_{d}+\varepsilon_{dk(i)} \right)^{T}Z_{d}(s_{i}) +W(s_{i}) \right)$ and assumed a Weibull baseline hazard i.e. $h_{0}\left( t_{j}(s_{i}) \right)=r({t_{j}(s_{i}) )}^{r-1}$ where $r$ is the shape parameter, $\boldsymbol{\beta}^{T}\boldsymbol{=}\left( \beta_{1}\boldsymbol{,\ldots,}\beta_{p} \right)$is the vector of regression coefficients with $\exp\left( \beta_{l} \right), l=1,\ldots p$, correspond to the hazard ratio (HR). $W(s_{i})$ is a cluster-specific random frailty which captures spatial correlation in mortality i.e. clusters in closer proximity are expected to have similar mortality hazard due to common exposures. We modeled $\boldsymbol{W}(s)={(W\left( s_{1} \right),W\left( s_{2} \right),\ldots,W\left( s_{m} \right))}^{T}$ by a Gaussian process, i.e. $\boldsymbol{W}(s)\sim N(0, )$, with an exponential correlation function of the distance $d_{kl}$ between locations $s_{k}$ and $s_{l}$, that is ${{}_{kl}=\sigma}^{2}exp(-d_{kl}\rho)$ [35]. The parameter $\sigma^{2}$ gives the variance of the spatial process and $\rho$ is a smoothing parameter that controls the rate of correlation decay with distance. For the exponential correlation function, $\frac{-log(0.05)}{\rho}$ determines the distance at which the correlation drops to $0.05$ (i.e. effective range of spatial process $(R)$). Our model assumed that the relation between health interventions and mortality varied across regions by including intervention specific spatially varying coefficients, $b_{d}+\varepsilon_{kd},$where $b_{d}$ is the association between intervention and mortality $d=1,\ldots D$ on child mortality at global (national) level and $\boldsymbol{\varepsilon}_{\boldsymbol{d}}\boldsymbol{=}\left( \varepsilon_{d1}\boldsymbol{,\ldots,}\varepsilon_{dk} \right)^{\boldsymbol{T}}$ are the varying effects at regional (sub-national) levels $k=1,\ldots K$ with $k\left( i \right)$ indicating the region $k$ corresponding to the location $s_{i}$.We introduced spatial dependence among the regions via a conditional autoregressive (CAR) prior for $\boldsymbol{\varepsilon}_{\boldsymbol{d}}$, that is $\boldsymbol{\varepsilon}_{\boldsymbol{d}}\sim N(\boldsymbol{0}, \Omega_{d})$ with $\Omega_{d}=\sigma_{d}^{2}\left( I-\gamma C \right)^{-1}\Delta. \sigma_{d}^{2}$ is the variance of spatially varying disease associations, $\Delta$ is a diagonal matrix with entries $\Delta_{kk}={g_{k}}^{-1}$where $g_{k}$ is the number of neighbors of region $k$, $\gamma$ measures overall spatial dependence and $C$ is a proximity matrix with normalized entries that is $C_{kl}=\omega_{kl}/g_{k}$, $\omega_{kl}$ is 1 if region $k$ neighbors $l$ and 0 otherwise [35]. To complete Bayesian model formulation, we assumed inverse gamma priors for all spatial variances with known parameters, i.e. $\sigma^{2}{,\sigma}_{d}^{2}\sim IG(2.01, 1.01)$, a uniform prior distribution for $\rho\sim U(a, b)$, where $a$ and $b$ chosen such as the effective range is within the maximum and minimum distances of the observed locations [36] and a uniform prior for $\gamma\sim U(\lambda_{1}^{-1},\lambda_{2}^{-1})$ where $\lambda_{1},\lambda_{2}$ are the smallest and largest eigenvalue of $\Delta^{\frac{-1}{2}}{C\Delta}^{\frac{1}{2}}$ [35]. The shape parameter was assigned an exponential prior $r\sim Exp(0.01)$. Non-informative normal priors were adopted for the regression coefficients $\beta_{l},b_{d}\sim N(0,{10}^{3})$ for $l=1,\ldots p$ and $d=1,\ldots D$.

The joint posterior distribution of the model is given by

$\prod_{\boldsymbol{i}} \boldsymbol{[} t_{j}(s_{i}) |\boldsymbol{\beta}\boldsymbol{,}b_{d}, W\left( s_{i} \right)\varepsilon_{dk},\boldsymbol{X}_{\boldsymbol{j}}\left( s_{i} \right),\boldsymbol{Z}_{\boldsymbol{d}}\left( s_{i} \right),r ]$[$\boldsymbol{W}(s)|\sigma^{2},\rho][\boldsymbol{\varepsilon}_{\boldsymbol{d}}\boldsymbol{|}\sigma_{d}^{2},\gamma][{\boldsymbol{\beta,}b_{d}, \sigma^{2},\sigma}_{d}^{2},\gamma,\rho,r]$.

Model parameters were estimated using Markov Chain Monte Carlo (MCMC) simulation [37]. We run a two chain algorithm for 250 000 iterations with an initial burn-in of 20,000 iterations. Convergence was assessed by visual inspection of trace and density plots and analytically by the Gelman and Rubin diagnostic [38].

**Bayesian variable selection**

A Bayesian stochastic search variable selection approach was used to determine the most important predictors of under-five mortality [39]. ITN coverage measures were highly correlated with more than 85%, therefore, only one (or none) ITN measure among those defining ownership and one (or none) ITN measure among those defining use [40,41] was selected. For each ITN coverage measure $X_{p}$ in the ownership group, a categorical indicator $I_{p}$, was introduced to represent exclusion of the variable from the model ($I_{p}=1$), or inclusion of one of the ITN ownership measure i.e. Prop_ITNA ($I_{p}=2$), Prop_1ITN2 ($I_{p}=3$) and Prop_1ITN ($I_{p}=4$). A similar definition was adopted for the ITN use coverage measure i.e. exclusion of the variable from the model ($I_{p}=1$), inclusion of Prop_ITN5 ($I_{p}=2$), Prop_ITNS ($I_{p}=3$) and Prop_ITNU ($I_{p}=4$). The ITN measure with the highest probability of inclusion in each category was included in the final model. For the environmental/climatic variables except land cover, variable selection compared their linear and categorical forms and selected the one that had the highest probability of inclusion or neither of the two forms. The categorical forms were generated based on the quartiles of variables. We introduced an indicator $I_{p}$ for each environmental/climatic covariate $X_{p}$ which defines exclusion of the variable from the model ($I_{p}=1$), inclusion in a categorical ($I_{p}=2$) or linear ($I_{p}=3$) form. For diseases, socio-demographic, land cover, health interventions other than ITN coverage measures, we introduced a binary indicator parameter $I_{p}$suggesting presence ($I_{p}=1$) or absence ($I_{p}=0$) of the predictor from the model.

$I_{p}$ has a probability mass function $\prod_{j=1}^{p} \pi_{j}^{\delta_{j}(I_{p})}$, where $\pi_{j}$ denotes the inclusion probabilities, $j=(1,2,3,4)$, e.g. for ITN coverage measures so that $\sum_{j=1}^{p} \pi_{j}=1$ and $\delta_{j}(.)$ is the Dirac function, $\delta_{j}(I_{p})=\left\{ \begin{aligned} 1, if I_{p}=j \\ 0, if I_{p}\neq j \end{aligned} \right.$. We assumed a spike and slab prior for regression coefficient $\beta_{p}$ corresponding to the corresponding covariate, $X_{p}$ i.e. $\beta_{p}\sim\left( 1-\delta_{1}{(I}_{p}) \right)N\left( 0,u_{0}\tau_{p}^{2} \right)+\delta_{1}(I_{p})N(0,\tau_{p}^{2})$ proposing a non-informative prior for $\beta_{p}$ in case $X_{p}$ is included in the model and an informative normal prior with variance close to zero (i.e. $u_{0}= {10}^{-3}$) shrinking $\beta_{p}$ to zero if $X_{p}$ is excluded from the model. For inclusion probabilities of ITN use and ownership, a non-informative Dirichlet distribution was adopted with hyper parameters $\alpha={(1,1,1,1)}^{T}$, that is, $\boldsymbol{\pi}={(\pi_{1},\pi_{2},\pi_{3},\pi_{4})}^{T}\sim Dirichlet(4,\alpha)$. A similar distribution was adopted for the inclusion probabilities of environmental/climatic factors. For diseases, socio-demographic, land cover, health interventions other than ITN coverage measures, a Bernoulli prior with an equal inclusion or exclusion probability was assumed for the indicator i.e. $I_{p}\sim bern(0.5)$. Also, inverse Gamma priors with parameters (2.01, 1.01) were assumed for the precision hyper parameters $\tau_{p}^{2}$. The predictors identified as important are those with posterior inclusion probability greater than or equal to 40% [40–42].
